# The Psoas Compartment Block for Hip Surgery: The Past, Present, and Future

**DOI:** 10.1155/2011/159541

**Published:** 2011-05-22

**Authors:** M. A. de Leeuw, W. W. A. Zuurmond, R. S. G. M. Perez

**Affiliations:** ^1^Department of Anesthesia, Intensive Care and Pain Medicine, Zaans Medical Centre, Koningin Julianaplein 58, 1502 DV Zaandam, The Netherlands; ^2^Department of Anesthesia, VU Medical Centre, 1502 DV Amsterdam, The Netherlands

## Abstract

A posterior lumbar plexus block or psoas compartment block (PCB) is an effective locoregional anesthetic technique for analgesia and anesthesia of the entire lower extremity including the hip. Since the first description in the early seventies, this technique has been modified based on advanced knowledge of the anatomical localization of the lumbar plexus and the improvement of technical equipment. This paper provides an overview of the history, clinical efficacy, and risk profile of the PCB focused on hip surgery. Current status and future expectations are discussed.

## 1. History

Although the principles of locoregional anesthesia were invented much earlier, it was Koller, an Austrian intern in ophthalmology, who introduced the first clinical locoregional anesthetic technique in 1884, using topical cocaine to the cornea for a glaucoma operation [1]. Five years later, the German surgeon Bier published [2], and his name became inseparably connected to the introduction of the first central neuraxis block, the spinal anesthesia. It subsequently took more than seven decades before the first description of a proximal lower extremity peripheral nerve block appeared. Winnie described an anterior approach for blocking the lumbar plexus [3]. The needle insertion point was just lateral to the femoral artery and 1 cm below the inguinal ligament. After paresthesia was elicited, more than 20 mL of local anesthetic was injected. Digital pressure below the needle insertion point was used to promote cephalad movement of the local anesthetic (within the femoral nerve sheath) purported to block the *three* main nerves of the lower extremity (femoral nerve, obturator nerve, and lateral femoral cutaneous nerve). In the same paper, the author briefly mentioned the possibility of a *posterior* lumbar paravertebral approach and presented this technique in a separate report one year later [4]. In 1976, Chayen et al. described a posterior approach of the lumbar plexus block named the *“Psoas Compartment Block” *(PCB) [5]. The anatomical compartment, formed by the psoas major muscle and its fascia on the anterior side, the transverse processes on the lateral side and the quadratus lumborum muscle on the posterior side, confines a space in which the lumbar plexus is located. Several years later, studies failed to confirm the existence of this so-called “psoas compartment” [6, 7]. Kirchmair et al. showed in a cadaver study that the lumbar plexus was situated *within *the psoas major muscle in the vast majority of specimens, and not *between* muscle and bony structures [7]. The last 4 decades, different approaches of the PCB have been proposed (Table 1). In 1989, Parkinson et al. described an L3 approach of the PCB (Dekrey's approach) [8] whereas Hanna et al. described an L2-L3 interspace approach of the PCB in 1993 [9]. Capdevila et al. modified the Winnie (L4) approach by a more medial needle insertion point compared to the Winnie approach [10]. Pandin in 2002 modified the Chayen approach with a more medial needle insertion point [11]. There were no significant differences in clinical efficacy between different approaches, but undesired side effects or even serious complications were described more often in the L3 approach and the approaches with a more medial needle insertion point [8, 10, 12–15]. Recently, Heller et al. showed in a cadaver study that except for the Pandin approach, other approaches were too lateral [16].

**Figure 1:**
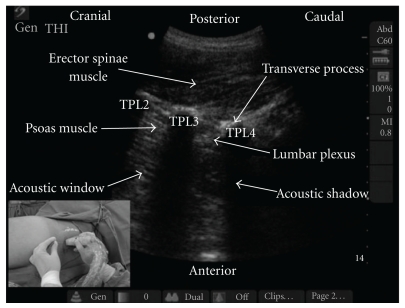
Longitudinal sonogram of the lumbar paravertebral region showing an optimal scan for lumbar plexus block. Picture in the inset shows the orientation of the ultrasound transducer and the direction in which the needle is introduced (long axis) during an ultrasound-guided lumbar plexus block. TP: transverse process. (picture used with permission from [20].

Parallel with the development of different approaches of the PCB, techniques to locate the lumbar plexus were also evolving. In 1974, Chayen et al. introduced the “loss of resistance” technique with a 20 ml syringe containing air [5]. In recent decades, nerve stimulation using a low-intensity current has become a common practice for locating the lumbar plexus [10–12, 17]. Furthermore, the use of ultrasound guidance has added value to the localization of the lumbar plexus [18–21]. Karmakar et al. described that parts of the lumbar plexus can be identified through the acoustic window of a longitudinal sonogram of the lumbar paravertebral region (Figure 1) [20]. Injected local anesthetics through a needle positioned close to the lumbar plexus could be followed under real-time ultrasound guidance producing an ipsilateral lumbar plexus block. Marhofer et al. described that at the L3–L5 level, the lumbar plexus, although deep, can be visualized using ultrasound [18]. However, the authors suggested the use of nerve stimulation in addition to ultrasound imaging to confirm the correct needle placement and recommended this combined technique as standard practice when performing a lumbar plexus block. Kirchmair et al. concluded that the efficacy of a PCB might be increased by ultasound guidance and that complications such as renal injury, that may occur during blind approaches, should be avoided by this technique [21]. 

## 2. Clinical Efficacy

Looking at the clinical efficacy, there is substantial evidence that a posterior approach of the lumbar plexus block has significant advantages compared to the anterior approach (femoral nerve block or “3-in-1 block”) of the lumbar plexus block. As the posterior approach is more effective in blocking the obturator nerve (the articular branches innervate the anteromedial capsule of the hip joint), the only real “3-in-1” block actually is the PCB [8, 22–24]. Biboulet et al. described lower visual analogue scale (VAS) scores during the first 4 h postoperative using a PCB compared with a femoral nerve block in patients undergoing a total hip arthroplasty [25]. To provide anesthesia and analgesia to the *entire *leg, a combination of a PCB and a “high” *sciatic nerve block *is necessary [5]. The addition of this sciatic nerve block to a PCB should also be valuable for hip surgery, because the posteromedial section of the hip joint capsule is partially innervated by branches of the sciatic nerve [26]. A PCB, with or without a sciatic nerve block, is of great value for postoperative *analgesia* after hip surgery. Different studies described a reduction of pain scores and a reduced consumption of rescue opioids after hip surgery due to the addition of a PCB [17, 25, 27, 28]. Stevens et al. described significant lower pain scores at *T* = 6 hours after total hip arthroplasty in patients receiving a single-injection posterior lumbar plexus block combined with general anesthesia, compared with patients who did not receive a PCB (VAS 1.4 ± 1.3 versus 2.4 ± 1.4, *P* = .007) [17]. Cumulative postoperative morphine consumption at *T* = 6 hours remained significantly lower as well (5.6 ± 4.7 mg versus 12.6 ± 7.5 mg, *P* < .0001) [17]. Biboulet et al. described the analgesic potency of a single-injection PCB compared with patient controlled analgesia (PCA) with intravenous morphine and a femoral nerve block (FNB) in patients undergoing a total hip arthroplasty [25]. At *T* = 4 hours after PCB, both VAS scores (1(0–2), 3(1.5–5.0), 2.5 (2–4) for, resp., PCB, FNB and PCA, data in median (IQR), *P* = .001) as well as morphine consumption (0 mg (0–6), 2 mg (0–16), 9 mg (0–18) for, respectively PCB, FNB, and PCA, data in median (IQR), *P* = .002 PCB versus PCA) were significantly lower in the PCB group [25]. In a meta-analysis, Touray et al. described that the reduction of pain of a single-injection PCB is limited to the first 8 hours after surgery [29]. This analgesic benefit may be extended beyond 8 hours by the use of a continuous infusion. Becchi et al. described the clinical efficacy of a continuous psoas compartment block after a total hip arthroplasty [27]. Low median pain scores at rest and after mobilization and less needed rescue analgesia during the whole study duration (48 hours) were described by the authors in the patients using the psoas catheter. A reduction of rescue opioids by the use of a continuous lumbar plexus block also has been described by Chelly et al. and Siddiqui et al. [30, 31]. Furthermore, Chudinov et al. described a significant reduction of pain scores during 32 hours after surgery by a continuous psoas compartment block in patients undergoing repair of a hip fracture [32]. Türker et al. described no significant differences in analgesic potency between a PCB and epidural analgesia for patients undergoing partial hip replacement surgery [33]. This implies a certain preference for a PCB as a postoperative analgesic strategy for hip surgery, because undesired side effects of epidural analgesia, such as urinary retention, hypotension, and pruritis, are avoided and the possibility of prolonged postoperative analgesia can be maintained [28, 34].

As sole *anaesthetic *technique for hip surgery, the PCB is likely to be insufficient. De Visme et al. described a substantial need for supplement opioids and sedatives for 27% of the patients undergoing hip fracture repair under PCB with an additional sacral plexus block [35]. Buckenmaier III et al. concluded that a lumbar plexus block with perineural catheter and sciatic nerve block with perioperative sedation is an effective alternative to general anesthesia for total hip arthroplasty [36]. However, the concentrations of propofol (50–200 mcg/kg/min) and fentanyl (327 ± 102 mcg) provided by the authors resemble general anesthesia instead of conscious sedation. A possible explanation of the insufficiency of the PCB as a sole anesthetic technique for hip surgery could be the variable innervation of the surgical site from the T12 and L1 dermatome, as described by Mannion et al. [28]. In a clinical efficacy study of PCB for prosthetic hip surgery, De Leeuw et al. concluded that a paravertebral block of L1 should be considered as additional technique to overcome the lack of anesthesia in dermatome L1 by a PCB [37]. 

## 3. Undesirable Side Effects and Complications

As with any other locoregional technique, a PCB has undesirable side effects. Seriously, even life threatening complications have been described in different case reports. In Table 2, undesirable side effects and complications of a PCB are pointed out [28].

The most frequently occurring side effect is the epidural diffusion of the injected local anesthetics. Reported incidences vary between 3 and 27% [25, 38]. A medial needle insertion point and a more cephalad lumbar approach (L2-L3) of the PCB seemed to be prognostic risk factors for this undesirable side effect [13, 14]. However, in a more recent publication, Mannion described that a large injected volume is probably the most important prognostic factor for bilateral spread, and not the approach of the PCB [28]. Another important factor which could influence the occurrence of epidural diffusion of local anesthetics after a PCB is the pressure during injection. Gadsden et al. concluded that injection of local anesthetic with high injection pressure (>20 psi) during lumbar plexus block commonly results in unwanted bilateral blockade and is associated with high risk of neuraxial blockade [39]. Retroperitoneal hematoma were described after either the performance of a single-injection PCB or the removal of a perineural psoas catheter [40, 41]. The majority of the hemorrhagic complications of a PCB were described in patients receiving anticoagulant or antiplatelet drugs, used for therapeutic indications or thromboprophylaxis [42]. Based on recent publications of large series of patients undergoing uneventful peripheral nerve blockade in combination with antithrombotic therapy as well as the case reports of hemorrhagic complications after peripheral nerve blocks, the American Society of Regional Anesthesia (ASRA) recommended that guidelines for anticoagulant and neuraxial blocks be applied for “deep” peripheral nerve blocks, like a PCB, including placement and removal of perineural catheters [42]. However, Chelly and Schilling described large series of uneventful continuous and single-injection lumbar plexus blocks, whereby catheters had been removed in anticoagulated patients without hemorrhagic complications, and questioned the evidence of the abovementioned ASRA recommendation [43, 44]. Renal subcapsular hematoma after an L3-PCB has been described by Aida et al. [15]. The inferior renal pole is close to the L3 level, therefore an L4 approach should be safer [13]. A feared complication of PCB is the inadvertent administration of local anesthetics in the intrathecal space leading to a total spinal anaesthesia. However, patients described in case studies by Pousman et al. and Gentili et al., where spinal anesthesia was reported to occur, were resuscitated without sequelae [45, 46]. The most serious complication of a PCB is the inadvertent intravascular injection of cardiotoxic local anaesthetics, rapidly leading to acute toxic reactions like seizures, cardiac arrest and eventually death [47]. Although false negative results are possible, the best way to prevent these acute toxic reactions remains aspiration prior injection, a negative test dose and a slow fractionated injection [13]. Treatment of a systemic cardiotoxic reaction consists of cardiopulmonary resuscitation and the infusion of intralipid [48]. A relatively highly serious complication rate of the PCB, compared to other lower limb peripheral nerve blocks, was described by Auroy et al. in a major French study [49]. Five serious complications after 394 PCB compared to none after 10309 femoral nerve blocks resulted in a substantial concern about this particular block [49]. These issues, possibly combined with more familiarity with alternative techniques such as neuraxial blocks, could be the reason for reluctance to the routine use of the PCB, leading to an underutilization of the PCB. 

## 4. Conclusions

In conclusion, PCB is proved to be effective as a locoregional technique for *analgesia* after hip surgery. Analgesic potency of a PCB is similar to epidural analgesia for hip surgery without the undesirable side effects. Further research is required to make PCB technique more optimal for anesthesia. In addition, the risk profile of the PCB should be evaluated more extensively. Until now, only one major study concerning complications of locoregional anesthetic techniques and some case reports concluded that a PCB has a relatively high risk profile. More elaborate (inter-)national PCB prospective complication registrations is therefore warranted. To reduce the risk of life-threatening complications, it is important to prevent the injection of large volumes of potentially cardiotoxic local anesthetics into the intrathecal space or into a blood vessel. Ultrasound imaging techniques could be helpful to optimize the needle position of this deep peripheral nerve block. To prevent a bilateral spread of local anesthetics after a PCB (the most frequent adverse effect), dose reduction (and therefore volume reduction), studies would be of great value. With regard to the risks and benefits of the PCB, further studies are required to evaluate the clinical efficacy of PCB in hip surgery and analyze the risk profile of this technique. 

## Figures and Tables

**Table 1:** Approaches of the lumbar plexus through the history.

Year	Author	Landmarks	Remarks
1974	Winnie	L4-L5; intersection line parallel spine through posterior superior iliac spine and intercristal line	Too lateral
1976	Chayen	L4-L5; 5 cm lateral and 3 cm caudal from spinous process L4	Too lateral
1989	Parkinson	L3; 3-4 cm lateral	L3 approach enhances the risk of renal puncture
1993	Hanna	L2-L3; 3–5 cm lateral	
2002	Capdevila	L4-L5; junction of lateral one third and medial two thirds of the line L4 and the line passes through posterior superior iliac spine (Modified Winnie approach)	Too lateral
2002	Pandin	L4-L5, 3 cm below intercristal line and 3 cm lateral to the interspinous line (Modified Chayen approach)	Too medial enhancing the risk of epidural spread of local anesthetics

**Table 2:** Undesirable side effects and complications of a PCB.

Epidural spread
Total spinal anesthesia
Mild hypotension
Plexopathy/Neuropathy
Systemic toxicity (central nervous system/cardiac)
Intraperitoneal injection
Retroperitoneal haematoma
Renal puncture
